# Exploring the Cytotoxic and Redox-Modulatory Effects of Nanoceria in MCF7 Breast Cancer Cells Using Integrated Molecular and Proteomic Analyses

**DOI:** 10.3390/antiox14111361

**Published:** 2025-11-14

**Authors:** Rukhsana Gul, Hicham Benabdelkamel, Mushtaq Ahmad Dar, Arwa Bazighifan, Afshan Masood, Salini Scaria Joy, Ousman Mahmood Ousman, Assim A. Alfadda

**Affiliations:** 1 Obesity Research Center, College of Medicine, King Saud University, P.O. Box 2925, Riyadh 11461, Saudi Arabia; helkamel@ksu.edu.sa (H.B.); oalothman@ksu.edu.sa (O.M.O.); aalfadda@ksu.edu.sa (A.A.A.); 2 Center of Excellence for Research in Engineering Materials (CEREM), Deanship of Scientific Research (DSR), King Saud University, Riyadh 11421, Saudi Arabia; mdar@ksu.edu.sa; 3 Strategic Centre for Diabetes Research, College of Medicine, King Saud University, Riyadh 11461, Saudi Arabia; sjoy@ksu.edu.sa; 4 Department of Medicine, College of Medicine, King Saud University, P.O. Box 2925, Riyadh 11461, Saudi Arabia

**Keywords:** nanoceria, breast cancer, oxidative stress, proteomics, apoptosis

## Abstract

Background: Cerium oxide nanoparticles (nanoceria) have attracted growing attention as promising anticancer agents due to their unique redox properties. Their selective cytotoxicity in cancer cells is thought to be mediated primarily through disruption of redox homeostasis. However, the precise molecular mechanisms underlying their action in breast cancer remain unclear. To address this gap, the present study investigates the dose-dependent cytotoxic, oxidative, and mitochondrial effects of nanoceria in MCF7 breast cancer cells, with mechanistic insights gained through gene expression and proteomic analyses. Methods: MCF7 breast cancer cells were treated with nanoceria (200 µg/mL and 400 µg/mL). Cytotoxicity, ROS levels, and mitochondrial membrane potential were assessed via MTT, DCFDA staining, and MitoTracker, respectively. Gene expression and label-free LC-MS/MS proteomics were used to evaluate molecular and pathway-level changes. Results: Nanoceria exhibited dose-dependent cytotoxicity, significantly reducing MCF7 cell viability to 61 ± 1.5% (*p* < 0.01) and 57 ± 1.8% (*p* < 0.01) at 200 µg/mL and 400 µg/mL, respectively, compared with the control. ROS levels increased 1.4-fold (*p* < 0.01) and 1.5-fold (*p* < 0.0001), accompanied by a decreased mitochondrial membrane potential by 11% (*p* < 0.01) and 25% (*p* < 0.05), indicating oxidative stress and mitochondrial dysfunction. Gene expression analysis supported activation of apoptotic pathways demonstrated by upregulation of *BNIP3*, the *BAX/BCL-2* ratio (*p* < 0.05), and disruption of mitochondrial homeostasis. Proteomic profiling revealed dose-specific alterations in >150 proteins (fold change ≥ 1.5, *p* < 0.05) related to redox balance, mitochondrial function, apoptosis, and cell cycle regulation. Conclusions: Nanoceria induces dose-dependent oxidative stress and mitochondrial dysfunction in MCF7 breast cancer cells, triggering apoptotic pathways and widespread alterations in protein expression. These results offer valuable mechanistic insights into nanoceria’s selective anticancer activity and highlight its potential as a promising therapeutic agent for breast cancer.

## 1. Introduction

Breast cancer remains the most frequently diagnosed malignancy and is one of the leading causes of cancer-related deaths globally [1,2]. According to the World Health Organization (WHO), breast cancer accounted for approximately 2.3 million new cases and 670,000 deaths globally in 2022, ranking it as the most common cancer among women and the second most cancer overall [2]. Similarly, data from the International Agency for Research on Cancer (IARC) reveal that breast cancer continues to represent the most prevalent cancer type among women and is the fourth leading cause of cancer death worldwide [3]. Epidemiological evidence indicates a steady rise in incidence rates across both developed and developing nations [4,5,6,7]. It remains a significant public health concern, particularly in transitional regions, which are experiencing a growing disease burden marked by rising incidence and sustained high mortality due to limited access to early detection or lack of advanced therapy.

Despite substantial progress in the extensive research on breast cancer and the introduction of advanced treatment strategies, including surgery, radiotherapy, chemotherapy, and hormone therapy, breast cancer still poses significant clinical challenges [8,9,10,11,12]. These include drug resistance, severe treatment-related side effects, tumor heterogeneity, and high recurrence rates. Global survival rates have improved significantly, with 5-year relative survival rates exceeding 80% in many countries, including a rate of 90% in the USA and 66.1% in India [13]. In comparison, survival remains lower in Saudi Arabia, rising only from 53.3% in 2010–2014 to 58.6% in 2020–2024 [14]. Treatment-related toxicity and the development of resistance to endocrine and chemotherapeutic agents continue to limit overall efficacy. Particularly in estrogen receptor-positive breast cancers, drug resistance continues to hinder long-term treatment success [15,16,17,18]. The MCF7 cell line derived from ER^+^ breast cancer serve as a well-established model in vitro for understanding the molecular mechanisms behind therapy resistance and identifying novel therapeutic targets [19].

In this context, nanotechnology has revolutionized cancer research by offering innovative tools for diagnosis, imaging, and therapy [20]. Among different nanomaterials, redox-sensitive material, capable of superoxide generation by targeting ROS signaling networks, triggers cancer cell apoptosis [21,22,23]. Over the last two decades, cerium oxide nanoparticles (nanoceria) have emerged as one of the most extensively studied rare earth nanomaterials, with remarkable potential in biomedical applications due to their unique physicochemical properties [24,25]. Nanoceria exhibit antioxidant, anti-inflammatory, antibacterial, antiviral, antifungal, and anticancer properties [26,27,28]. Their distinct redox-switching ability between Ce^3+^ and Ce^4+^ states allows them to mimic the enzymatic activities of superoxide dismutase (SOD), catalase (CAT), and oxidase thereby enabling regulation of intracellular reactive oxygen species (ROS) levels [29]. Depending on the surrounding pH and oxidative environment, nanoceria can act as either pro-oxidant or antioxidant, promoting oxidative damage in the acidic tumor microenvironment (pH 6.4) while preserving normal cells under physiological conditions (pH 7.4) [30,31,32]. Owing to this unique redox property, several in vivo and in vitro studies have explored the application of nanoceria for the treatment of various cancers [33,34,35].

ROS generation and regulation play a dual role in cancer biology [21]. At moderate concentrations, ROS function as signaling molecules that regulate proliferation and survival; however, at elevated levels they trigger DNA damage, protein oxidation, and lipid peroxidation via the induction of oxidative stress, ultimately triggering apoptosis. Nanoceria’s ability to disrupt redox homeostasis in cancer cells through elevated ROS production and interference with mitochondrial function makes them promising redox active agent for cancer therapy. Their surface chemistry and auto-regenerative redox cycling enable sustained biological activity, a feature lacking in conventional chemotherapeutics. Despite these promising features, the detailed molecular mechanisms underlying nanoceria-induced cytotoxic effects on breast cancer cells remain poorly understood. In this study, we investigate the dose-dependent effects of nanoceria (200 µg/mL and 400 µg/mL) on the human ER^+^ MCF7 breast cancer cell line using a combination of molecular assays and quantitative proteomics. We hypothesized that nanoceria would trigger oxidative stress-driven mitochondrial dysfunction, leading to altered gene and protein expression, disruption of key signaling pathways, and reduction in cancer cell survival. By integrating these approaches, we aim to provide a comprehensive understanding of the mechanism by which nanoceria modulate redox balance and cellular homeostasis in MCF7 cells, contributing to their potential development as a targeted therapeutic approach for breast cancer.

## 2. Materials and Methods

### 2.1. Cell Culture

Breast cancer cells (MCF7) were obtained from ATCC and grown in DMEM (ATCC 30-2002) supplemented with 10% FBS (ATCC 30-2020) and penicillin/streptomycin (100 U/mL and 100 mg/mL). Cells were maintained in culture medium at 37 °C in a humidified atmosphere with 5% CO_2_. For experimentation, they were plated in 6-well plates at a density of 1 × 10^5^ cells per well. By the following day, the cultures reached approximately 70–80% confluence, at which point assays were carried out. For the proteomic studies, MCF7 cells were sub-cultured in T75 flasks and divided into three groups (1) untreated control, (2) nanoceria (200 μg/mL), and (3) nanoceria (400 μg/mL), with n = 4 in each group. Cells were pre-treatment with nanoceria at different time points depending on the experiment and then trypsinized and lysed to isolate proteins, following previously established protocols [26].

### 2.2. MTT Assay for Assessing Cell Toxicity

Cell viability was assessed by MTT assay (Promega, Madison, WI, USA) [36]. In order to determine cell toxicity, cells were plated in 96-well plates and allowed to adhere overnight before treatment with varying concentrations of nanoceria (100, 200, or 400 µg/mL). In live cells, the yellow MTT is converted within the mitochondria into insoluble purple formazan. This conversion occurs only in the presence of active mitochondrial reductase enzymes, making the reaction directly proportional to the number of viable cells. Following 24, 48, and 72 h of incubation, 20 µL of MTT reagent was added to each well. The plates were subsequently incubated for one more hour, after which the absorbance was recorded at 570 nm using a microplate reader (BioTek Synergy, Winooski, VT, USA).

### 2.3. ROS Production Calculation

The amount of ROS was determined using fluorometry and a cell-permeable redox-sensitive dye 2,7-dichlorodihydrofluorescein diacetate DCFH-DA reagent in the control and treatment groups as described [26]. DCFDA dye (4 μm) was added to the samples for 30 min, cells were washed twice with DPBS and incubated at 37 °C for 30 min. Nanoceria at concentrations of 50, 100, 200, and 400 µg/mL was added to medium containing 2% FBS, and the plates were incubated at 37 °C for 30 min. Measurements were performed using a Synergy HT multi-mode 96-well plate reader (BioTek Instruments, Inc., Winooski, VT, USA) at an excitation wavelength of 488 nm and emission wavelength of 520 nm.

### 2.4. RNA Isolation and Quantitative Real-Time Polymerase Chain Reaction

RNA extraction was performed with TRIzol (Invitrogen, Carlsbad, CA, USA) according to methods described earlier [37]. Cells were lysed in TRIzol, and RNA was isolated following chloroform separation and centrifugation. Nanodrop (Thermo Scientific™ NanoDrop 2000c, Waltham, MA, USA) was used to measure the concentration and purity of RNA. cDNA was synthesized from 400 ng RNA using SuperScript™ III (Invitrogen, Carlsbad, CA, USA). Real-time PCR was performed using SYBR Green mix (Applied Biosystems, Waltham, MA, USA) on a 7500 Real-Time PCR System. Thermal cycling was carried out under standard conditions. β-actin was used as an internal control, and relative expression was calculated using the 2^−ΔΔCt^ method. Primer sequences, procured from Macrogen Inc. (Seoul, South Korea), are provided in Supplementary Table S1.

### 2.5. Measurement of Mitochondrial Potential

Mitochondrial membrane potential was evaluated using Mito Tracker Green and Mito Tracker Red (Applied Biosystems, Waltham, MA, USA), as described earlier [37]. MCF7 cells were seeded in 12-well plates and exposed to nanoceria (200 µg/mL and 400 µg/mL) for 24 h. Following treatment, cells were stained with 100 nM MitoTracker Green and 500 nM MitoTracker Red (Applied Biosystems, Waltham, MA, USA) for 30 min at 37 °C each. Nuclei were counterstained with Hoechst 33342 (Santa Cruz Biotechnology, Dallas, TX, USA). After washing with PBS, DMEM medium was added to each well, and images were captured using a live-cell imager (Floid Cell Imaging Station, Thermo Fisher Scientific, Waltham, MA, USA). The relative mitochondrial membrane potential was determined by calculating the ratio of Mito Tracker Red to Mito Tracker Green fluorescence intensities. A decrease in red fluorescence indicates membrane depolarization.

### 2.6. Sample Preparation for Proteomics

#### 2.6.1. Protein Extraction and Digestion

Protein extraction from the MCF7 cell line was carried out using a modified version of a previously published protocol [37]. Briefly, 2 million cells were harvested and resuspended in 200 µL lysis buffer (pH 8.8) containing 30 mM Tris-HCl, 7 M urea, 2 M thiourea, and 2% CHAPS, 1× protease inhibitors. The suspension was agitated vigorously for 2 min, followed by an incubation period of one hour at 4 °C with continuous orbital shaking at 450 rpm. To ensure complete cell disruption, the homogenate was sonicated (Microsonicator, Qsonica, Newtown, CT, USA) using a 30% pulse setting for three interval cycles with a 1-min cooling pause. The crude lysate was then centrifuged at 16,000× *g* for 15 min at 4 °C. The cleared supernatant was treated with 4 volumes of ice-cold acetone and stored at −20 °C overnight for protein precipitation. The precipitated protein material was collected by centrifugation at 14,000× *g* for 15 min, the supernatant was decanted, and the remaining protein pellets were solubilized in 2× concentration of lysis buffer. The 2D-Quantkit was used to assess protein concentrations of all samples in triplicate (GE Healthcare, Piscataway, NJ, USA) [27,36].

Fifty micrograms of proteins were used for further experiments. Proteins were suspended in 10 µL of denaturing buffer containing 6 M urea. Disulfide linkages were cleaved by adding 1 µL of dithiothreitol (200 mM) and incubated for 30 min at 60 °C. The exposed sulfhydryl groups were then capped by alkylation by adding 1 µL of 400 mM iodo-acetamide solution and incubated for an additional 30 min in the absence of light at room temperature. For tryptic digestion, the samples were diluted with 65 µL of ammonium bicarbonate buffer (50 mM), followed by adding 2.5 µL of sequencing grade modified trypsin (Promega Corporation, Madison, WI, USA) (1 µg/μL). Hydrolysis was carried out by overnight incubation at 37 °C, and the reaction was quenched by adding 7 µL of 10% formic acid. The purification and desalting of peptides were carried out using a commercially available kit (Pierce C18 spin columns, Thermo Scientific, Waltham, MA, USA), and the peptide concentration was subsequently assessed using a Pierce Quantitative Colorimetric Peptide Assay (Thermo Scientific, Waltham, MA, USA). Finally, eluted peptides were evaporated to complete dryness using vacuum centrifugation (Eppendorf Concentrator plus^TM^, Hamburg, Germany).

#### 2.6.2. Peptide Processing and Liquid Chromatography

Dried peptide residues were dissolved in 0.1% (*v*/*v*) formic acid medium [38]. One microliter of each sample was introduced into a Dionex UltiMate 3000 series (DIOnex Softron GmbH, Germering, Germany) featuring a WPS-3000 autosampler for automated injection. The peptides were concentrated on a PepMap100 C18 trap column (3 µm, 100 Å, 75 µm i.d. × 20 mm, nanoViper; Thermo Scientific, Rockford, IL, USA) that was pre-equilibrated with an aqueous mobile phase with 0.05% trifluoroacetic acid (TFA).

#### 2.6.3. Peptide Separation

Following sample trapping, peptides were separated on a PepMap™ C18 analytical, Thermo Scientific, Rockford, IL, USA column (50 cm × 75 μm) at a flow rate of 300 nL/min. The chromatographic separation was performed using a mobile phase consisting of two components. Mobile phase A was an aqueous solution containing 0.1% *v*/*v* formic acid in water, and mobile phase B consisted of 0.1% *v*/*v* formic acid and 80% *v*/*v* acetonitrile in water. The elution schedule was executed as a gradient elution, performed as follows: the column was initially pre-equilibrated with 5% mobile phase B, followed by a linear increase to 22.5% mobile phase B over 139 min. The gradient was further increased to 45% mobile phase B over the next 45 min, for a total run time of 184 min.

#### 2.6.4. Mass Spectrometry Analysis

Eluted peptides were subjected to analysis using a Q Exactive Plus Hybrid Quadrupole-Orbitrap mass spectrometer (Thermo Fisher Scientific, Waltham, MA, USA) operating in positive ion mode. The instrument was equipped with a nanospray ion source with a potential of 2000 V. Mass spectra were collected using the data-dependent acquisition (DDA) mode with a maximal duty cycle time of 3 s. The primary mass spectrometry (MS) scan range was set from 375 to 1650 *m*/*z* with a scanning resolution of 70,000. Tandem mass spectrometry (MS/MS) scans were initiated from a minimum of *m*/*z* of 80 *m*/*z* with a resolution of 17,500. Dynamic exclusion was set to 20 s. Automatic gain control (AGC) targets were set to 3 × 10^6^ for MS scans and 1 × 10^5^ for MS/MS scans [39].

### 2.7. Data Processing

Data are expressed as mean ± standard error of the mean (SEM), unless otherwise indicated. For evaluating differences among experimental groups, one-way analysis of variance (ANOVA), followed by Tukey’s post hoc test for multiple pairwise comparisons were used. To compare two independent groups, an unpaired two-tailed Student’s *t*-test was chosen. Proteomics data were analyzed using the statistical functions in Proteome Discoverer v3.0, with group comparisons performed using a built-in Student’s *t*-test. Multiple testing correction was applied, and proteins with false discovery rate (FDR)-adjusted *p*-values (q-values) < 0.01 were considered significant. Differentially expressed proteins were defined by two criteria: FDR-adjusted *p* ≤ 0.05 and a fold change ≥ 1.5. The significantly regulated proteins were exported from Proteome Discoverer for further analysis. Pathway enrichment and functional analysis were performed using Ingenuity Pathway Analysis (IPA; QIAGEN, Hilden, Germany, http://www.ingenuity.com), which integrates the experimental dataset with known biological interactions from curated literature to identify key pathways and networks. In addition, protein classification based on molecular function and biological processes was carried out using the PANTHER system (http://www.pantherdb.org, accessed 25 June 2025) Across all analyses in this study, a *p*-value of <0.05 was taken as the threshold indicating statistically significance.

## 3. Results

### 3.1. Cytotoxic and Pro-Oxidant Effects of Nanoceria on MCF7 Breast Cancer Cells

**Figure 1:**
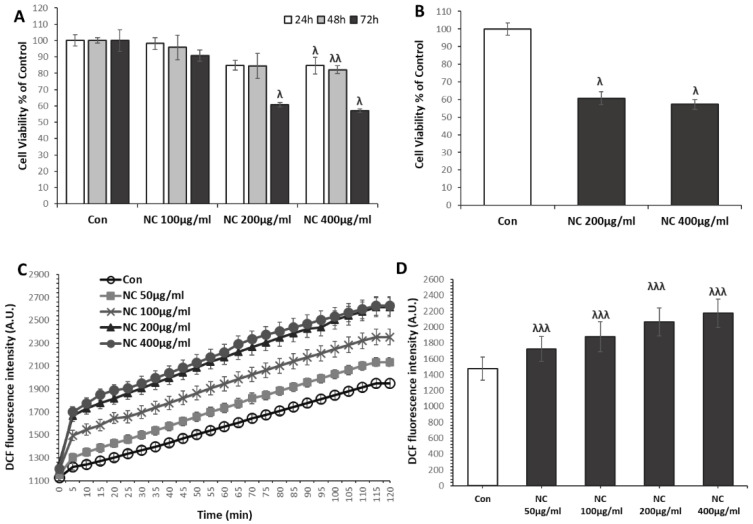
Nanoceria (NC) induces dose- and time-dependent cytotoxicity and promotes ROS generation in MCF7 cells. (**A**) MCF7 cells were treated with increasing concentrations of NC (100, 200, and 400 µg/mL) for 24, 48, and 72 h. (**B**) To highlight the difference between the two most effective concentrations, 200 µg/mL and 400 µg/mL, data were compared separately to illustrate the concentration-dependent variation in cytotoxic response. (**C**,**D**) Intracellular ROS production was measured using the DCFDA assay in a kinetic manner over 2 h following nanoceria treatment (50, 100, 200, and 400 µg/mL). ROS levels increased significantly in a dose-dependent fashion compared to untreated controls, with the highest induction observed at 400 µg/mL. Data are presented as mean ± SD from at least three independent experiments. ^λ^ *p* < 0.05, ^λλ^ *p* < 0.01, ^λλλ^ *p* < 0.001 vs. control.

To evaluate the cytotoxic effects of nanoceria, MCF7 cells were treated with varying concentrations (100 µg, 200 µg, and 400 µg) and incubated for different durations (24, 48, and 72 h). The viability of the cells was assessed using the MTT assay. Nanoceria markedly reduced cell viability in both a dose- and time-dependent fashion (Figure 1A,B). Meanwhile, 100 µg/mL of nanoceria showed a trend towards reduced viability, but it was not significant. On the other hand, both 200 and 400 µg/mL treatments significantly reduced cell viability, with 400 µg/mL demonstrating a more pronounced effect at all durations. To further confirm these observations, cell viability was assessed using the trypan blue exclusion assay, which showed a comparable decline at 48 and 72 h (Supplementary Figure S1A). The results further demonstrate a clear dose-dependent cytotoxic effect, with 400 µg/mL exerting the strongest impact compared to 200 µg/mL (Supplementary Figure S1A).

To determine whether oxidative stress contributed to the observed cytotoxicity, intracellular ROS levels were measured using the DCFDA assay. As illustrated in Figure 1C,D, ROS generation was monitored in real time over a 2 h kinetic period. Nanoceria-treated cells showed a clear dose-dependent increase in ROS production compared with untreated controls. The highest ROS induction was observed at 400 µg/mL nanoceria, followed by 200, 100, and 50 µg/mL. A significant elevation in ROS levels was observed at all tested concentrations throughout the 2 h time course.

### 3.2. Nanoceria Downregulates Antioxidant Defense and Induces Apoptosis in MCF7 Cells

**Figure 2:**
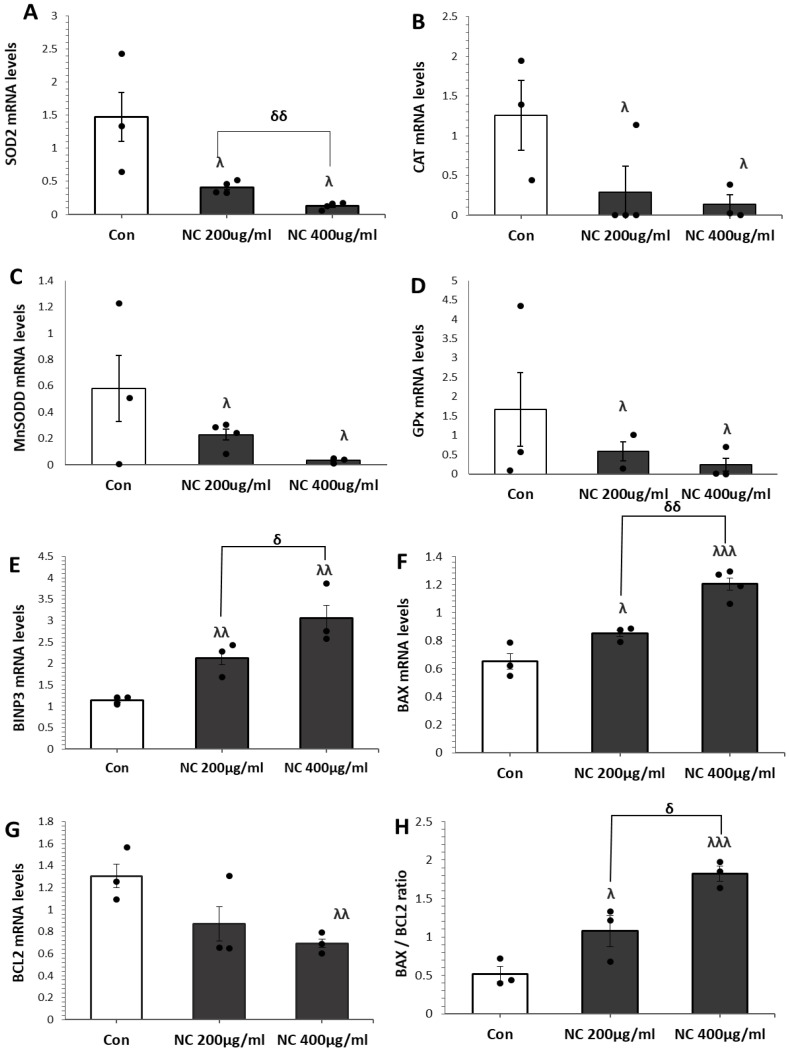
Nanoceria (NC) downregulates the expression of antioxidant defense genes in MCF7 cells. MCF7 cells were treated with NC at concentrations of 200 and 400 µg/mL for 24 h, and the mRNA expression of antioxidant genes, *SOD2* (**A**), *CAT* (**B**), *MnSOD* (**C**), and *GPx* (**D**), as quantified by qRT-PCR. Relative gene expression levels were normalized to β-actin and expressed as fold change compared to untreated controls. MCF7 cells were treated with NC at concentrations of 200 and 400 µg/mL for 24 h. The mRNA expression levels of *BNIP3* (**E**), *BAX* (**F**), and *BCL-2* (**G**) were quantified by qRT-PCR, and the *BAX*/*BCL-2* ratio (**H**) was subsequently calculated. Data are presented as mean ± SD of three independent experiments. ^λ^ *p* < 0.05, ^λλ^ *p* < 0.01, ^λλλ^ *p* < 0.001 vs. control; ^δ^ *p* < 0.05, ^δδ^ *p* < 0.01 vs. 200 µg/mL group (Student’s *t*-test).

We further investigated the effects of nanoceria on the expression of genes involved in the antioxidant defense system using quantitative real-time PCR (qRT-PCR). The expression levels of *CAT*, *SOD2*, manganese superoxide dismutase (*MnSOD*), and glutathione peroxidase (*GPx*) were significantly reduced in cells exposed to 200 and 400 µg/mL nanoceria compared to untreated controls (Figure 2A–D).

To further investigate the mechanism underlying nanoceria-induced cell death, we analyzed the mRNA expression of key apoptotic regulators (*BNIP3*, *BAX*, and *BCL-2*), using qRT-PCR following treatment with 200 and 400 µg/mL nanoceria. Treatment with nanoceria resulted in significant upregulation of the pro-apoptotic genes *BNIP3* and *BAX*, accompanied by a marked downregulation of the anti-apoptotic gene *BCL-2* (Figure 2E–H). These effects were more pronounced at 400 µg/mL compared to 200 µg/mL. Additionally, the *BAX/BCL-2* ratio, a marker of apoptotic propensity, was significantly increased in nanoceria-treated cells, further supporting the induction of apoptosis. Furthermore, *NF-κB* levels were also significantly elevated upon nanoceria treatment compared to the control (Supplementary Figure S1B), suggesting potential involvement of inflammatory or stress-related pathways. These results demonstrate that nanoceria weakens the antioxidant defense system and shifts the balance toward apoptosis in MCF7 cells.

### 3.3. Nanoceria Induces G1 Phase Arrest via p53–p21 Pathway in MCF Cells

**Figure 3:**
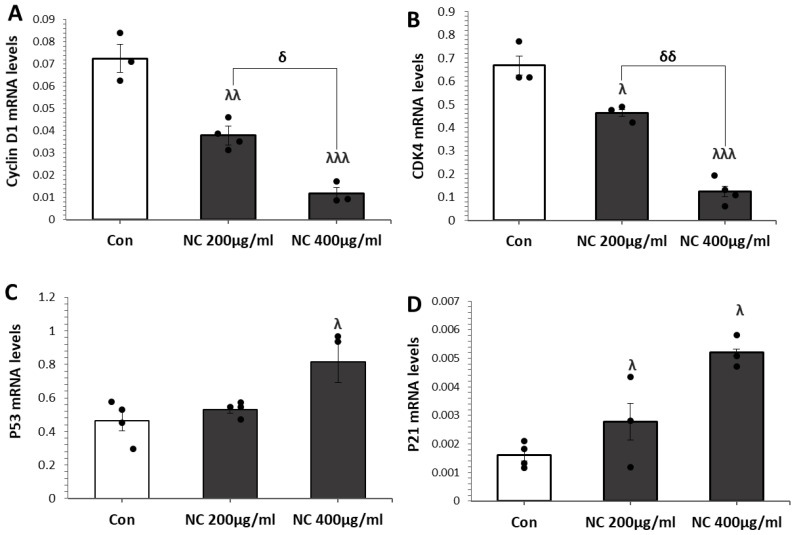
Nanoceria (NC) modulates cell cycle regulatory gene expression in MCF7 cells. MCF7 cells were treated with 200 and 400 µg/mL NC, and the mRNA expression levels of *cyclin D1*, *CDK4*, *p53*, and *p21* were analyzed using qRT-PCR. NC treatment significantly downregulated *cyclin D1* (**A**) and *CDK4* (**B**), while upregulating *p53* (**C**) and *p21* (**D**) in a concentration-dependent manner, showing greater effects at 400 µg/mL. Data are presented as mean ± SD from three independent experiments. ^λ^ *p* < 0.05, ^λλ^ *p* < 0.01, ^λλλ^ *p* < 0.001 vs. control; ^δ^ *p* < 0.05, ^δδ^ *p* < 0.01 vs. 200 µg/mL group.

To elucidate the impact of nanoceria on cell cycle regulation, we quantified the mRNA expression of critical cell cycle regulatory genes (*cyclin D1*, *CDK4*, *p53*, and *p21*) in MCF7 cells using qRT-PCR (Figure 3A–D). Treatment with 200 and 400 µg/mL nanoceria significantly downregulated the expression of *cyclin D1* and *CDK4* at both concentrations, with a more pronounced suppression observed at 400 µg/mL. Conversely, nanoceria treatment led to a marked upregulation of the tumor suppressor *p53* and its downstream effector *p21*, again showing dose-dependent enhancement. These results suggest that nanoceria induces G1 phase arrest in MCF7 cells by downregulating cyclin D1/CDK4 and activating the p53–p21 pathway, consistent with a stress response to nanoceria exposure.

### 3.4. Nanoceria Inhibits Mitochondrial Biogenesis and Induces Mitochondrial Dysfunction in a Dose-Dependent Manner in MCF7 Cells

**Figure 4:**
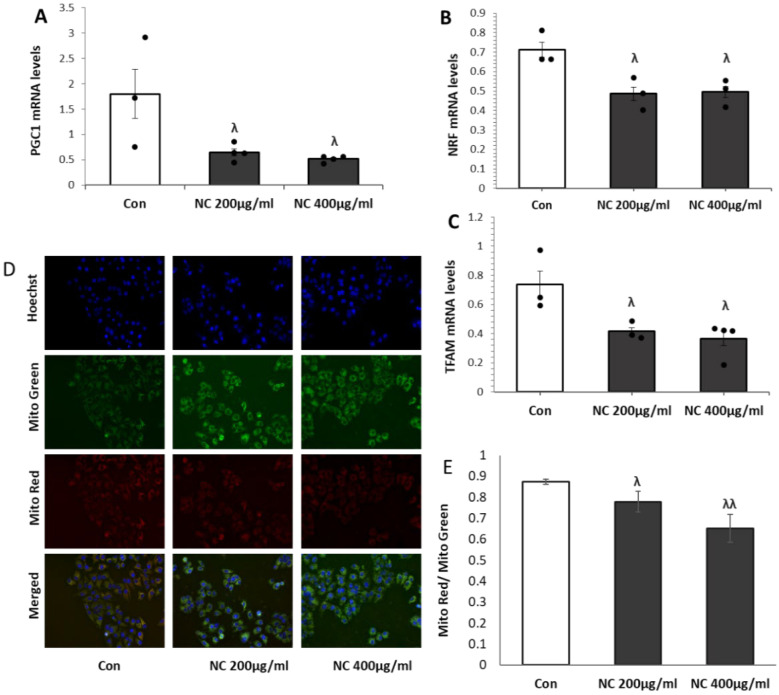
Nanoceria suppresses mitochondrial biogenesis and disrupts mitochondrial function in MCF7 cells. (**A**–**C**) Relative mRNA expression levels of *PGC-1α*, *NRF1*, and *TFAM* were measured by qRT-PCR in MCF7 cells treated with 200 and 400 µg/mL nanoceria. (**D**) Assessment of mitochondrial membrane potential using MitoTracker Red/Green staining. (**E**) A decrease in the red/green fluorescence intensity ratio indicates mitochondrial depolarization. Data are expressed as mean ± SEM from three independent experiments. ^λ^ *p* < 0.05, ^λλ^ *p* < 0.01 vs. control.

Proper regulation of mitochondrial biogenesis is key for preserving mitochondrial integrity and function. To evaluate the impact of nanoceria on mitochondrial biogenesis, we assessed the mRNA expression of key regulatory genes (*PGC-1α*, *NRF1*, and *TFAM*) in MCF7 cells treated with 200 and 400 µg/mL nanoceria using qRT-PCR. Nanoceria treatment led to a significant, dose-dependent reduction in *PGC-1α* expression compared to control levels (Figure 4A). Similarly, *NRF1* and *TFAM* transcripts were markedly suppressed following treatment (Figure 4B,C), indicating an inhibitory effect on mitochondrial biogenesis. In parallel, analysis using the MitoTracker Red/Green fluorescence ratio revealed a significant decline in mitochondrial membrane potential upon nanoceria exposure, with the maximum reduction observed at 400 µg/mL, suggesting impaired mitochondrial function (Figure 4D,E).

### 3.5. Label-Free Quantitative Proteomics Analysis

Label-free quantitative proteomics was conducted to determine a comparative analysis of the three groups (untreated control, 200 µg/mL and 400 µg/mL nanoceria-treated samples). The Total Ion Chromatogram Mass Spectrometry (TIC MS) revealed distinct profiles for each group (control and 200 µg/mL and 400 µg/mL nanoceria-treated samples), indicating variations in abundance and distribution across the retention time (Supplementary Figure S2). Key peaks and overall TIC intensities varied between the groups, suggesting differential protein expression patterns. After removing missing values, 5698 proteins were consistently identified across all three experimental groups (Supplementary Table S1).

### 3.6. Analysis of Differentially Expressed Proteins in Untreated Control and 200 µg/mL and 400 µg/mL Nanoceria-Treated Samples

**Figure 5:**
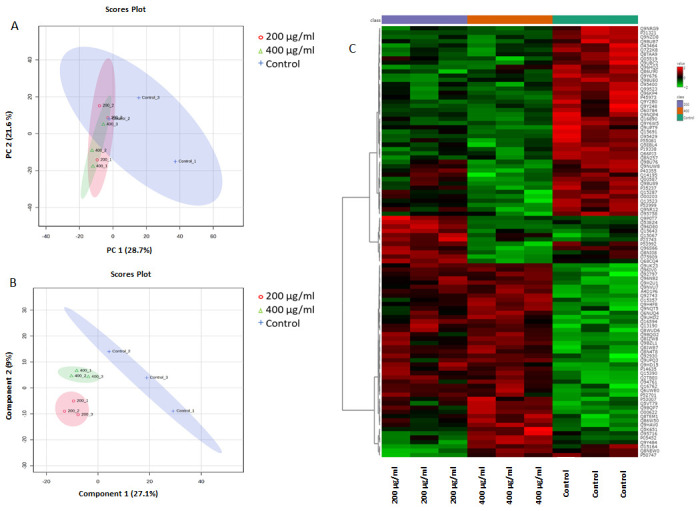
Multivariate analysis of proteomic profiles of untreated control and 200 µg/mL and 400 µg/mL nanoceria-treated samples. (**A**) PCA displays semi-separation between the untreated control and the 200 µL and 400 µL nanoceria-treated groups. (**B**) PLS-DA revealed a semi-distinction between the untreated control and the 200 µg/mL and 400 µg/mL nanoceria-treated samples. (**C**) HAC and heat map visualization were used to examine proteins that were significantly altered between the untreated control and the 200 µg/mL and 400 µg/mL nanoceria-treated samples. The color scale represents protein regulation, with green indicating downregulated proteins and red indicating upregulated proteins.

The proteins that distinguished between the three groups are displayed in Figure 5. The visualization of each study group and outlier detection were carried out using principal component analysis (PCA). Figure 5 shows two-dimensional PCA score plots of the three groups, with clear separation between the untreated control and the 200 µg/mL and 400 µg/mL nanoceria-treated groups, indicating distinct proteomics profiles and significant differences in protein expression patterns across treatments. To identify distinguished proteomic changes between the groups, a PLS-DA model was used to identify the candidate proteins with the highest potential to discriminate between the untreated control and the 200 µg/mL and 400 µg/mL nanoceria-treated samples (Figure 5B). The heat map (Figure 5C) effectively represents the top 100 proteins, showing significant differences among the untreated controls and the samples treated with 200 µg/mL and 400 µg/mL nanoceria. These proteins may serve as indicators of the molecular, biological, and cellular alterations induced by varying nanoceria concentrations.

### 3.7. Protein Dysregulation in Response to Altered Nanoceria Treatment Concentration

**Figure 6:**
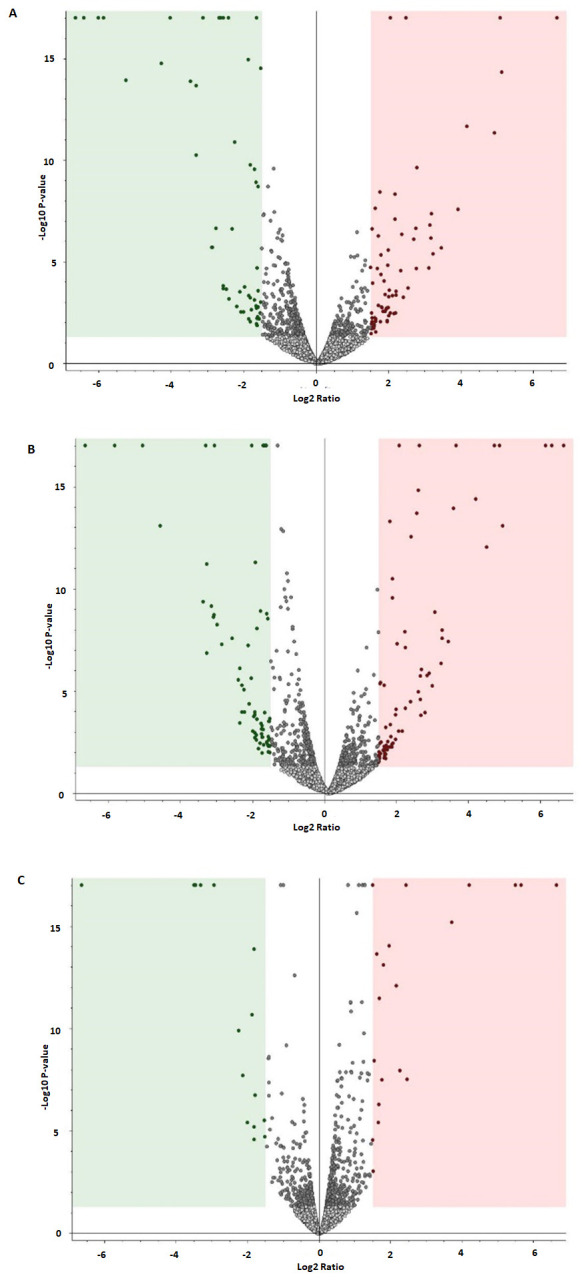
Volcano plot revealing significant alterations in the abundance of multiple proteins: (**A**) 200 µg/mL nanoceria-treated samples vs. untreated control; (**B**) 400 µg/mL nanoceria-treated samples vs. untreated control; and (**C**) 400 µg/mL nanoceria-treated samples vs. 200 µg/mL nanoceria-treated samples. The green dots represent downregulated and red dots represent upregulated proteins in groups. The grey dots represent statistically non-significant proteins (unpaired *t*-test, FDR *p*-value ≤ 0.05, fold change ≥ 1.5).

Volcano plot analysis was conducted to identify significantly dysregulated proteins following nanoceria treatment. Significantly altered proteins were identified by a relative abundance fold change exceeding 1.5 or falling below 0.66, along with a *p*-value of less than 0.05. As depicted in Figure 6A, treatment with 200 µg/mL nanoceria resulted in the significant dysregulation of 348 proteins when compared to untreated control samples. Of these, 145 proteins showed significant upregulation (indicated in red), while 203 proteins were significantly downregulated (indicated in green). The comparison of the 400 µg/mL nanoceria-treated samples to untreated controls (Figure 6B) revealed 345 significantly dysregulated proteins. Specifically, 189 proteins were found to be upregulated (red), and 156 proteins were downregulated (green). Analysis of protein expression between the 400 µg/mL and 200 µg/mL nanoceria-treated samples (Figure 6C) identified 155 significantly dysregulated proteins. In this comparison, 111 proteins demonstrated upregulation (red), and 44 proteins showed downregulation (green).

**Figure 7:**
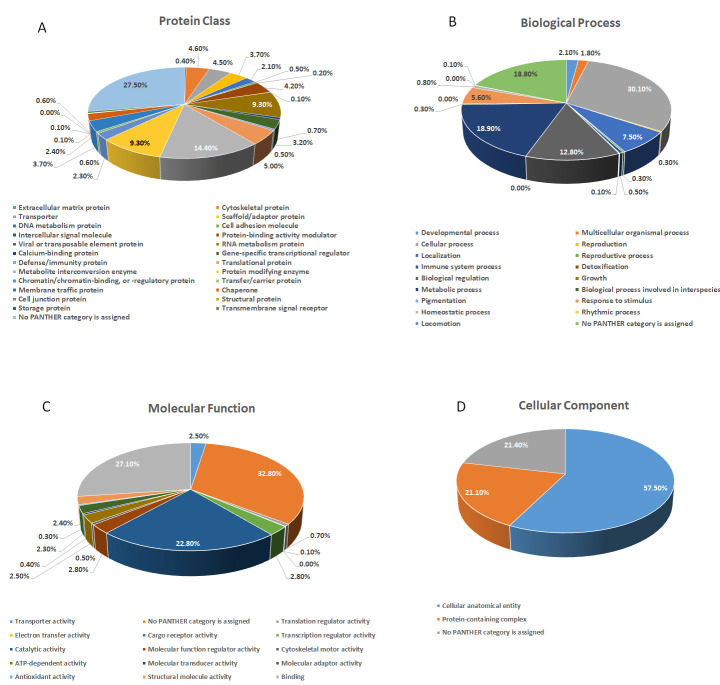
Comparative depiction of identified proteins categorized into groups according to their protein class (**A**), biological process (**B**), molecular functions(**C**), and cellular components (**D**)**.**

The PANTHER (Protein Analysis Through Evolutionary Relationships) classification system was applied to categorize the identified proteins based on their protein classes (Figure 7A), biological processes (Figure 7B), molecular functions (Figure 7C), and cellular components (Figure 7D). In protein class majority of proteins identified were metabolite interconversion enzyme (14.4%), protein modifying enzyme (9.3%), and RNA metabolism protein (9.3%) (Figure 7A). Regarding biological processes, the proteins were mainly associated with cellular processes (30.1%), metabolic processes (18.9%), and biological regulation (12.8%) (Figure 7B).Functional categorization revealed that the majority of differentially expressed proteins were enzymes, with a predominant role in binding (27.01%), followed by catalytic activity (22.8%) (Figure 7C). The majority of the identified proteins were located in the cellular anatomical entity (57.5%), followed by the protein containing complex (21.1%) (Figure 7D).

### 3.8. Interaction Network Analysis of Differentially Expressed Proteins

**Figure 8:**
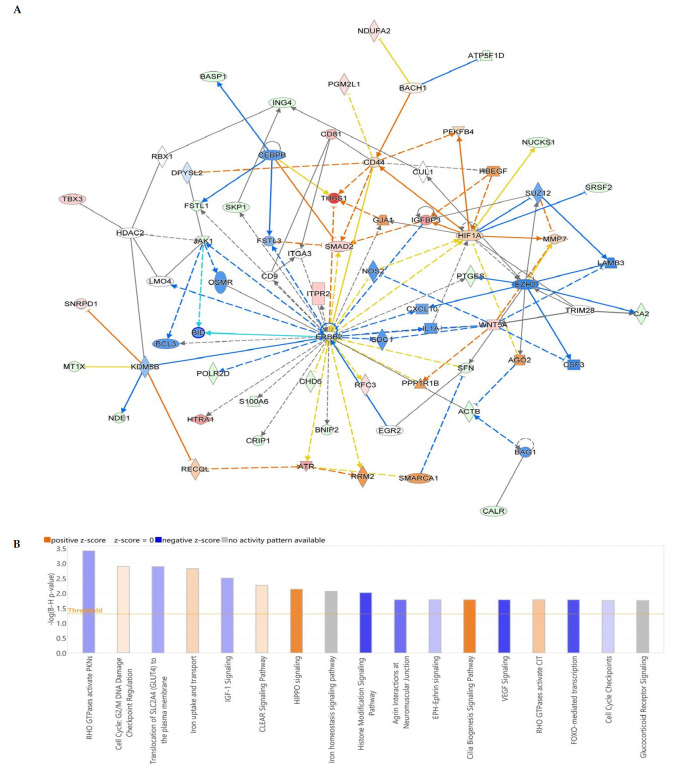
(**A**) Network pathways derived from differentially regulated proteins in nanoceria-treated (200 µg/mL) versus untreated control samples. Green nodes indicate downregulated proteins, and red nodes indicate upregulated proteins. Solid lines: Represent direct physical interactions between molecules (e.g., protein-protein interaction). Dashed/dotted lines: Represent indirect functional or regulatory relationships (e.g., one molecule affects the expression or activity of another through one or more intermediate molecules not shown in that specific view). (**B**) The most significant canonical pathways ranked by *p*-value, which were used to construct interaction networks is shown. The blue bars represent negative z scores, the orange bars represent positive z scores, and the gray bars indicate that no activity pattern was available. The interaction networks were generated through IPA (QIAGEN, Inc.).

The biological consequences of the observed changes in protein abundances were analyzed using Ingenuity Pathway Analysis (IPA) software version 9.0 (Ingenuity Systems, Redwood, CA, USA). IPA calculates a score by fitting the input dataset of proteins against its curated biological functions database to generate protein–protein interaction networks. For comparison of 200 µg/mL nanoceria-treated samples versus untreated controls, IPA identified cellular development, cellular growth and proliferation, and organismal injury and abnormalities as the top network pathways, with the highest network score of 34 (Figure 8A). The top canonical pathways included histone modification signaling pathways, VEGF signaling, and FOXO-mediated transcription (all with negative z-scores), while HIPPO signaling and cilia biogenesis signaling pathways (both withpositive z-scores) (Figure 8B). The IPA identified cell death and survival, cellular growth and proliferation, organismal injury and abnormalities as the most affected network pathways, with the highest score of 27 observed in 400 µg/mL nanoceria-treated samples vs. untreated controls (Supplementary Figure S3A). The top canonical pathways at this comparison included the processing of capped intron containing pre-mRNA(negative z-score), and cellular response to heat stress, RHO GTPases activating formins, and RHO GTPases activating CIT(positive z-scores) (Supplementary Figure S3B). The IPA identified cell cycle, cell death and survival, and cellular movement as the most affected network pathways, with the highest score of 43 in 400 µg/mL nanoceria-treated samples vs. untreated controls (Supplementary Figure S4A). The top canonical pathways included regulation of endogenous retroelements, senescence-associated secretory phenotype, histone modification signaling pathway, etc. with a positive z-score (Supplementary Figure S4B).

## 4. Discussion

Our study demonstrates that nanoceria exerts dose-dependent cytotoxic and oxidative stress-inducing effects in MCF7 breast cancer cells. Both the tested concentrations (200 µg/mL and 400 µg/mL) induced oxidative stress, mitochondrial damage, and disrupted redox homeostasis. However, our data demonstrate that the higher dose (400 µg/mL) depicted more pronounced effects compared to a concentration of 200 µg/mL, suggesting a dose-dependent effect. A disruption was observed in several cellular signaling processes, including mitochondrial integrity and cell cycle progression, ultimately leading to a reduction in cell viability. Proteomic data corroborated these findings, revealing alterations in the expression of proteins that are involved in mitochondrial metabolism, oxidative stress response, apoptosis, and cell cycle regulation. Together, these findings highlight the potential of nanoceria as a redox-active, dose-sensitive therapeutic agent in breast cancer treatment.

We observed a dose-dependent reduction in MCF7 cell viability upon nanoceria exposure, accompanied by a significant rise in ROS levels. This confirms oxidative stress is a key mediator of nanoceria-induced cytotoxicity. While nanoceria is widely described for its antioxidant properties in non-cancerous or normal physiological models, our findings reveal a contrasting behavior in breast cancer cells [31]. At higher concentrations (200-400 µg/mL), nanoceria enhances ROS generation beyond the antioxidant capacity of MCF cells, resulting in oxidative stress-mediated cytotoxicity [22,32].

This paradoxical behavior of nanoceria is largely attributed to its auto-regenerative redox cycling between the Ce^3+^ and Ce^4+^ oxidation states, which is strongly influenced by the intracellular pH environment. Therefore, owing to its ability to change oxidation states, nanoceria has a dual nature and can function either as an antioxidant or pro-oxidant. In normal cells, which maintain a near-neutral intracellular pH (7.2), nanoceria tends to maintain a higher Ce^3+^/Ce^4+^ ratio and mimics antioxidant enzymes like SOD and catalase, effectively scavenging superoxide radicals and hydrogen peroxide. Perez et al. demonstrated that nanoceria coated with dextran exhibited antioxidant and cytoprotective effects under physiological pH, efficiently neutralizing ROS and protecting normal cells from oxidative injury. However, this protective capacity diminishes under acidic conditions [40]. Conversely, cancer cells, including MCF7 breast cancer cells, typically exhibit a mildly acidic extracellular pH (6.4–7) and slightly alkaline intracellular pH (7.4–7.6) due to altered metabolic reprogramming and increased proton efflux [41,42]. The higher pH favors the stabilization of the Ce^4+^ state, which enhances its pro-oxidant nature and reduces its antioxidant potential. Consistent with this, Tian et al. reported that a pH-responsive polymer–nanoceria hybrid exhibited potent catalytic ROS generation and cytotoxicity in acidic tumor-like environments, whereas it retained antioxidant activity at neutral pH, minimizing damage to normal tissues [43]. Consequently, nanoceria induces ROS generation in MCF7 cells rather than scavenging, thereby inducing oxidative damage, mitochondrial dysfunction, and cell cycle arrest [44]. Therefore, the redox behavior of nanoceria is context-dependent, and it acts as a cytoprotective antioxidant in normal tissues but switches to a cytotoxic pro-oxidant in the acidic environment of cancer cells, offering a mechanistic rationale for its selective anticancer effects.

Recent studies have demonstrated that the basal ROS concentration is higher in cancer cells than normal cells, which promotes cancer cell proliferation and contributes to therapy resistance because of an overactivated antioxidant defense system, which protects cancer cells [45,46]. Additionally, it is well known that excessive ROS generation can overwhelm the antioxidant defense system and damage cells due to oxidative stress [47]. Therefore, cancer researchers are looking for ways to boost ROS generation inside cancer cells or disturb their redox balance, as cancer cells are highly vulnerable to ROS-induced damage [48]. In agreement with this concept, we noted a significant downregulation of antioxidant defense genes, such as *SOD2* and *GPX1*, both of which are crucial for neutralizing superoxide radicals and hydrogen peroxide, respectively. Their diminished expression may render MCF7 cells more vulnerable to oxidative stress induced by nanoceria, which further shift the redox balance towards a pro-oxidant state. This downregulation of antioxidant machinery not only contributes to sustained ROS levels but also amplifies mitochondrial and genomic damage. Our findings are consistent with earlier studies demonstrating the redox-dependent cytotoxicity of nanoceria in cancer models [31,49,50] Similar to our observations in MCF7 cells, Abedi et al. reported increased ROS generation and mitochondrial dysfunction as key mechanisms underlying nanoceria-induced apoptosis [50]. Moreover, studies on other cancer types, such as lung and colon cancers, have shown that nanoceria selectively exerts pro-oxidant effects in tumor cells while preserving normal cell viability through its dual redox nature [32,51].

This oxidative imbalance is further supported by mitochondrial dysfunction, as evidenced by the decrease in mitochondrial membrane potential. Loss of mitochondrial membrane potential is an early indicator of mitochondrial depolarization and intrinsic apoptotic signaling [52]. Our findings demonstrate that exposure of MCF7 cells to nanoceria induces excessive ROS generation, loss of mitochondrial membrane potential, and upregulation of pro-apoptotic markers, indicating that mitochondrial dysfunction precedes apoptosis. Elevated ROS directly impair mitochondrial components, triggering the opening of mitochondrial permeability transition pores, outer membrane rupture, and activation of intrinsic apoptotic pathways [22,53]. These results suggest that mitochondrial dysfunction is a causal event rather than a consequence of apoptosis. Studies using cancer cells have demonstrated that mitochondrial dysfunction arising from oncogenic stress leads to disrupted energy metabolism, altered redox signaling, and enhanced ROS production, which can preferentially impact tumor cells [54]. High ROS levels initiate intrinsic apoptotic signaling via mitochondrial pathways by downregulating anti-apoptotic BCL-2 proteins and promoting cytochrome c release, which ultimately results in programmed cell death [55,56].

Furthermore, nanoceria-induced apoptosis in MCF7 cells appears to be mediated at least in part through the upregulation of p53 and its downstream effects on mitochondrial integrity. The observed increase in p53 gene expression and increase in ROS generation, along with a loss of mitochondrial membrane potential, suggest a coordinated mechanism in which oxidative stress and p53 signaling act synergistically to promote apoptotic cell death. This interpretation is supported by some earlier reports, where p53 serves as a key regulator of mitochondrial dynamics and metabolism, balancing survival and death of cells through its control of oxidative stress responses and apoptotic signaling [57]. Consistent with our data, other studies have shown that nanoparticle exposure can induce ROS-dependent p53 activation, leading to mitochondrial dysfunction and apoptosis in cancer cells [58,59]. Therefore, nanoceria-induced apoptosis in MCF7 cells appears to be driven by a coordinated interplay between oxidative stress, p53-mediated mitochondrial signaling, and ROS-induced mitochondrial dysfunction, highlighting mitochondrial redox imbalance as a potential therapeutic target in cancer treatment.

In line with this, the observed downregulation of genes involved in mitochondrial biogenesis and transcriptional regulation points toward a nanoceria-induced suppression of mitochondrial functions. This suppression may impair ATP production, redox balance, and overall cellular resilience, compounding the cytotoxic effects. In parallel, cell cycle analysis revealed a marked arrest at the G0/G1 phase, suggesting activation of checkpoint mechanisms in response to oxidative and genotoxic stress. Importantly, we observed a significant upregulation of tumor suppressor protein p53 and its downstream effector p21, both central to the DNA damage response. The p53/p21 axis is known to mediate G1 arrest by inhibiting cyclin-CDK complexes, allowing the cell time to repair damage or enter apoptosis if repair fails. Our findings imply that nanoceria activates this pathway through ROS-mediated DNA damage and mitochondrial signaling, reinforcing its role in halting cancer cell proliferation.

To gain broader insight into the molecular perturbations, we employed label-free quantitative proteomics, which identified multiple differentially expressed proteins involved in key cellular processes. Cells exposed to 400 µg/mL nanoceria displayed differential expression of numerous proteins involved in redox homeostasis, apoptosis regulation, DNA damage response, and cell cycle control. The proteomics data supported our transcriptional findings, showing downregulation of mitochondrial proteins and antioxidant enzymes and upregulation of stress-related proteins. Proteins related to oxidative phosphorylation, redox homeostasis (e.g., peroxiredoxins, thioredoxins), and mitochondrial structural maintenance were significantly affected, highlighting mitochondria as a primary target of nanoceria action. Moreover, proteomic changes aligned with the observed cell cycle arrest, with modulation of proteins associated with cyclin regulation, DNA repair, and checkpoint control, further corroborating the p53-p21-mediated arrest mechanism. Several of these changes mirror hallmarks of mitochondria-dependent apoptosis and stress-induced senescence, suggesting that nanoceria not only halts cell cycle progression but may also steer cells toward irreversible growth arrest or programmed cell death depending on dosage and exposure duration.

The gene expression and proteomic datasets collectively indicate a consistent response to nanoceria treatment in MCF7 cells. The downregulation of antioxidant defense genes accompanied byreduced expression of corresponding proteins indicates that nanoceria disrupts redox homeostasis. Similarly, the transcriptional activation of p53 and p21 corresponded with proteomic data, indicating modulation of cell cycle checkpoints and apoptotic proteins that lead to cell death. The inference of mitochondrial dysfunction from alterations in gene expression was corroborated by proteomic enrichment of pathways associated with oxidative phosphorylation, mitochondrial integrity, and apoptosis. This strong cross-validation between gene and protein data emphasizes that nanoceria cytotoxic effects are primarily mediated through redox imbalance, mitochondrial impairment, and the activation of intrinsic apoptotic signaling pathways. Our proteomic analysis further complements these findings by revealing modulation of oxidative phosphorylation, redox-regulating enzymes, and apoptotic signaling proteins, providing molecular evidence for nanoceria’s selective anticancer activity.

## 5. Conclusions

**Figure 9:**
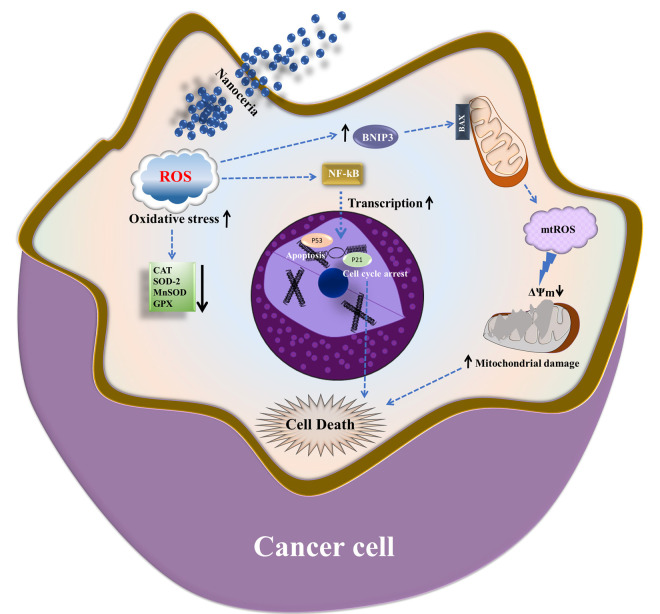
Proposed mechanism of nanoceria-induced cytotoxicity in MCF7 breast cancer cells. Nanoceria triggers excessive ROS production, activating NF-κB and pro-apoptotic signaling (p53, p21), leading to cell cycle arrest and apoptosis. Elevated ROS and BNIP33 expression promote mitochondrial dysfunction, mtROS accumulation, loss of membrane potential (ΔΨm), and suppression of antioxidant enzymes, leading to cancer cell death.

In conclusion, our study reveals that nanoceria induces a mitochondria-centric cascade of interrelated cellular events in MCF7 breast cancer cells, initiated by excessive ROS generation and antioxidant system collapse, leading to mitochondrial dysfunction, suppression of mitochondrial biogenesis, p53 pathway activation, G0/G1 cell cycle arrest, and global proteomic reprogramming. This integrated response reflects a disruption of redox balance and energetic stress that ultimately compromises cancer cell viability (Figure 9). The concentration-dependent nature of these effects, with the most pronounced effect at 400 µg/mL, highlights the importance of surpassing a critical oxidative threshold to achieve therapeutic efficacy. By perturbing the redox environment, mitochondrial integrity, and proteostasis of cancer cells, nanoceria emerges as a promising candidate for nanoparticle-based anticancer strategies. However, further studies into its selectivity, optimal dosing, and long-term effects are warranted.

### Strengths and Limitations

The major strength of our study is the integration of gene expression and proteomic data, which collectively establish a link between oxidative stress, mitochondrial dysfunction, and cell cycle arrest in MCF7 cells by nanoceria treatment. Additionally, we tested the dose-dependent effect of nanoceria treatment, which demonstrated concentration-specific cellular responses in MCF7 cells. However, a limitation is the absence of comparison with a non-cancerous mammary epithelial cell line as a control, which would have further validated the selectivity of nanoceria cytotoxicity. Therefore, future studies will be planned to evaluate the effects of nanoceria on cancer cells compared to normal cells, and in vivo models will be employed to substantiate the translational relevance of our findings.

## Data Availability

All relevant data are within the manuscript and its Supplementary Materials.

## Supplementary Materials

The following supporting information can be downloaded at https://www.mdpi.com/article/10.3390/antiox14111361/s1.
