# Supplementary material for: Exploring the Cytotoxic and Redox-Modulatory Effects of Nanoceria in MCF7 Breast Cancer Cells Using Integrated Molecular and Proteomic Analyses
Source: Antioxidants (Basel). 2025 Nov 14;14(11):1361. doi: 10.3390/antiox14111361 (PMC12649274; doi:10.3390/antiox14111361)
Supplement: Supplementary file 1 [file antioxidants-14-01361-s001.zip › antioxidants-3905507-supplementary.pdf]

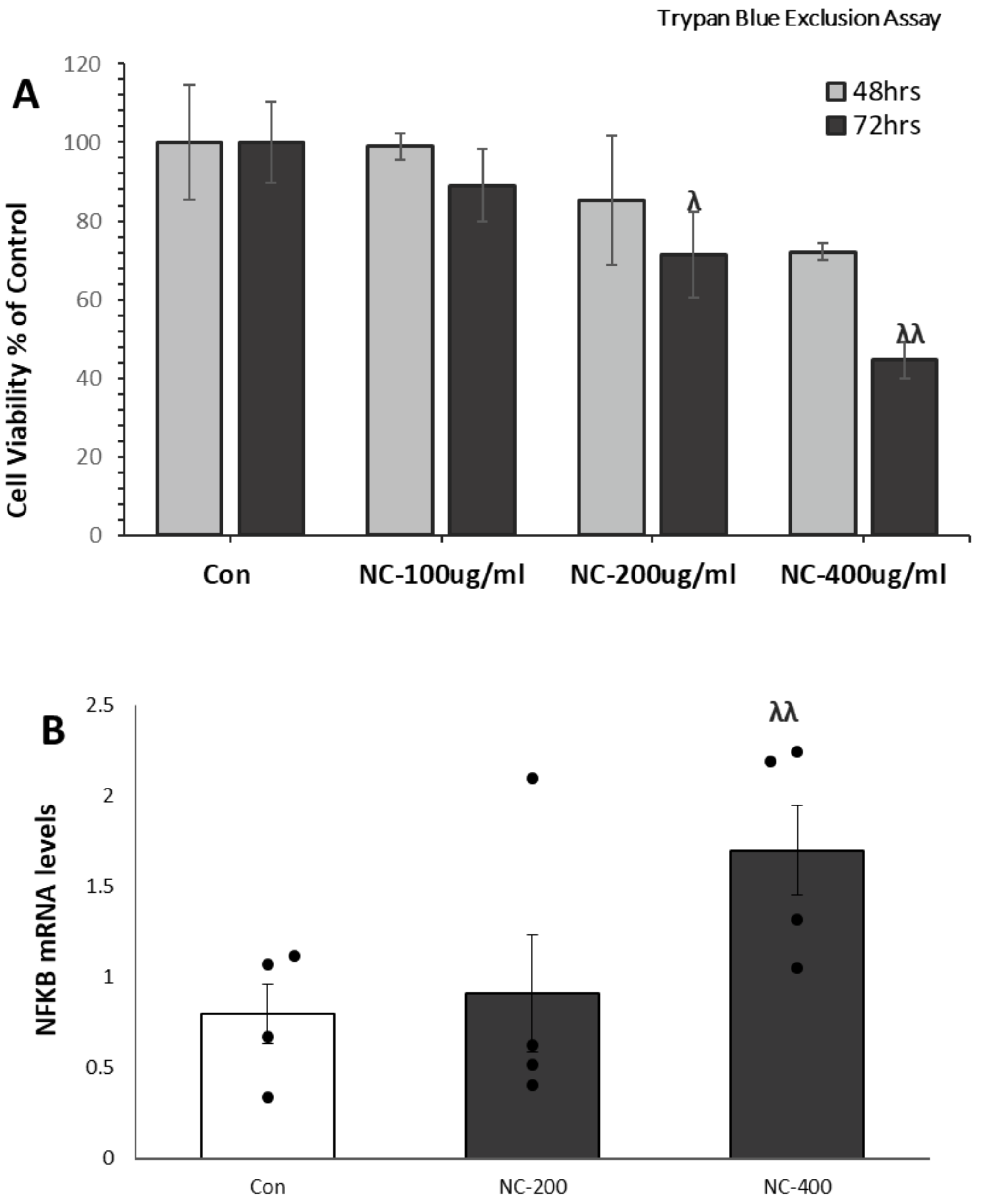

Supplementary Figure S1. (A) Trypan blue exclusion assay showing dose-dependent reduction in MCF7 cell viability after 48 and 72 h of nanoceria treatment. (B) NF-κB expression in MCF7 cells treated with 200 and 400 μg/mL nanoceria. NF-κB levels were significantly higher in cells treated with 400 μg/mL compared to control and 200 μg/mL.λp < 0.05, λλp < 0.01 vs. control.

A

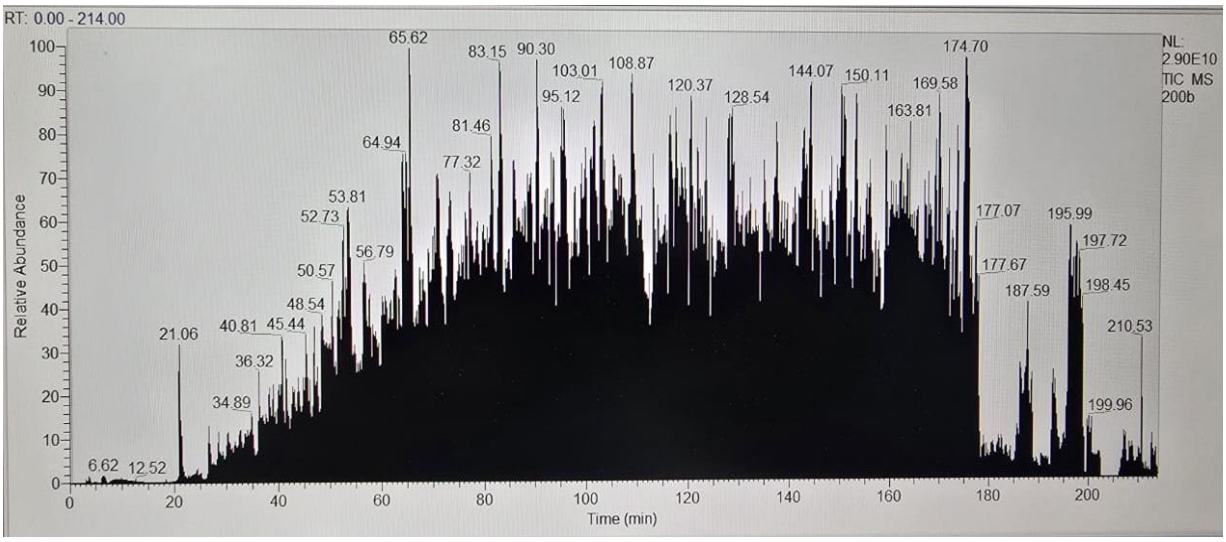

B

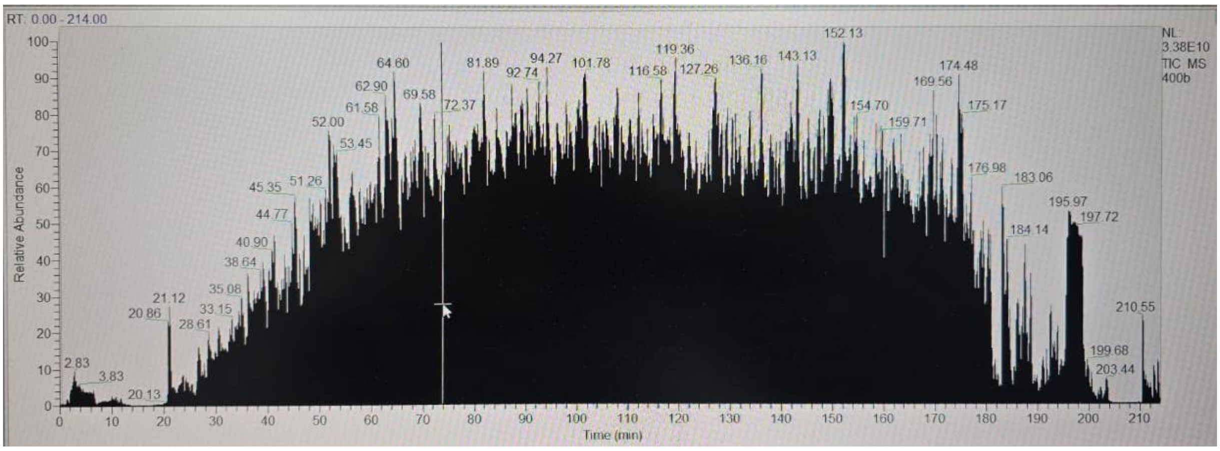

C

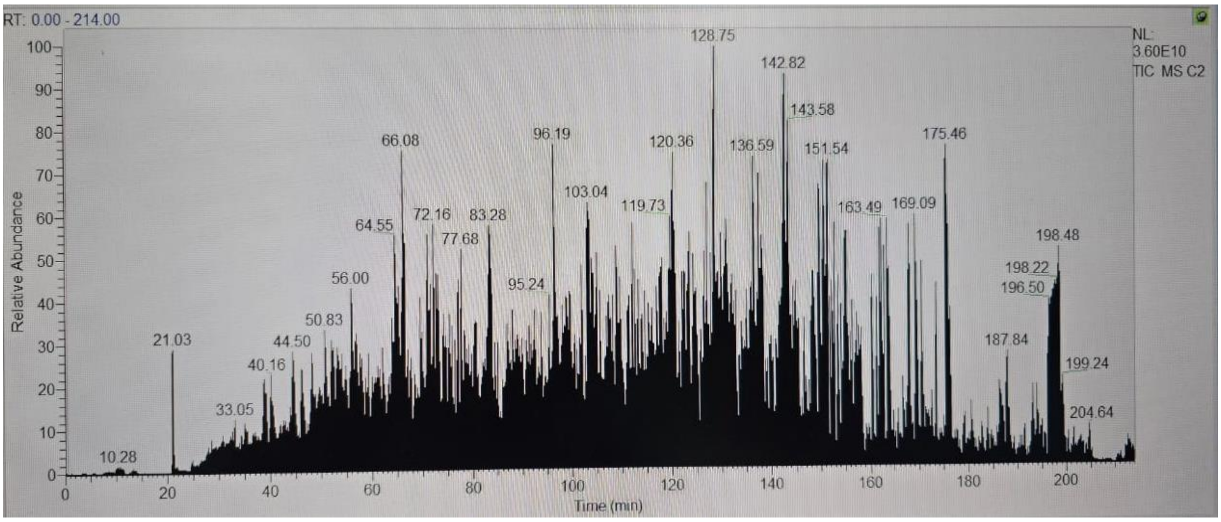

Supplementary Figure S2. The figure is a representative of the total ion chromatograms of MCF7 cells (A) untreated control, (B) cells treated with 200 μg/ml nanoceria, and (C) cells treated with 400 μg/ml nanoceria.

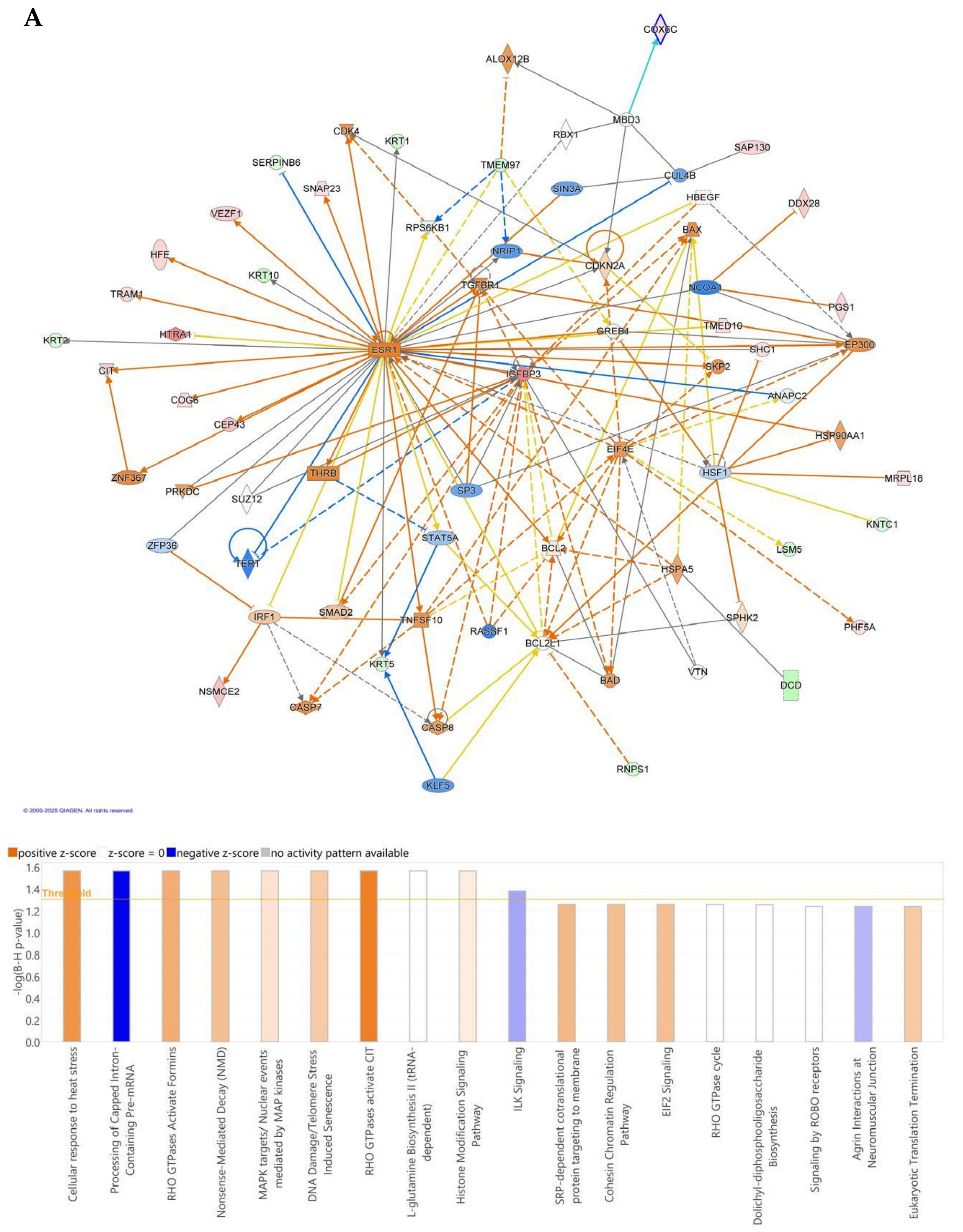

Supplementary Figure S3. (**A**) Network pathways derived from differentially regulated proteins in nanoceria-treated (400 μg/mL) versus untreated control samples. Green nodes indicate downregulated proteins, and red nodes indicate upregulated proteins. (**B**) The most significant canonical pathways ranked by p-value, which were used to construct interaction networks.

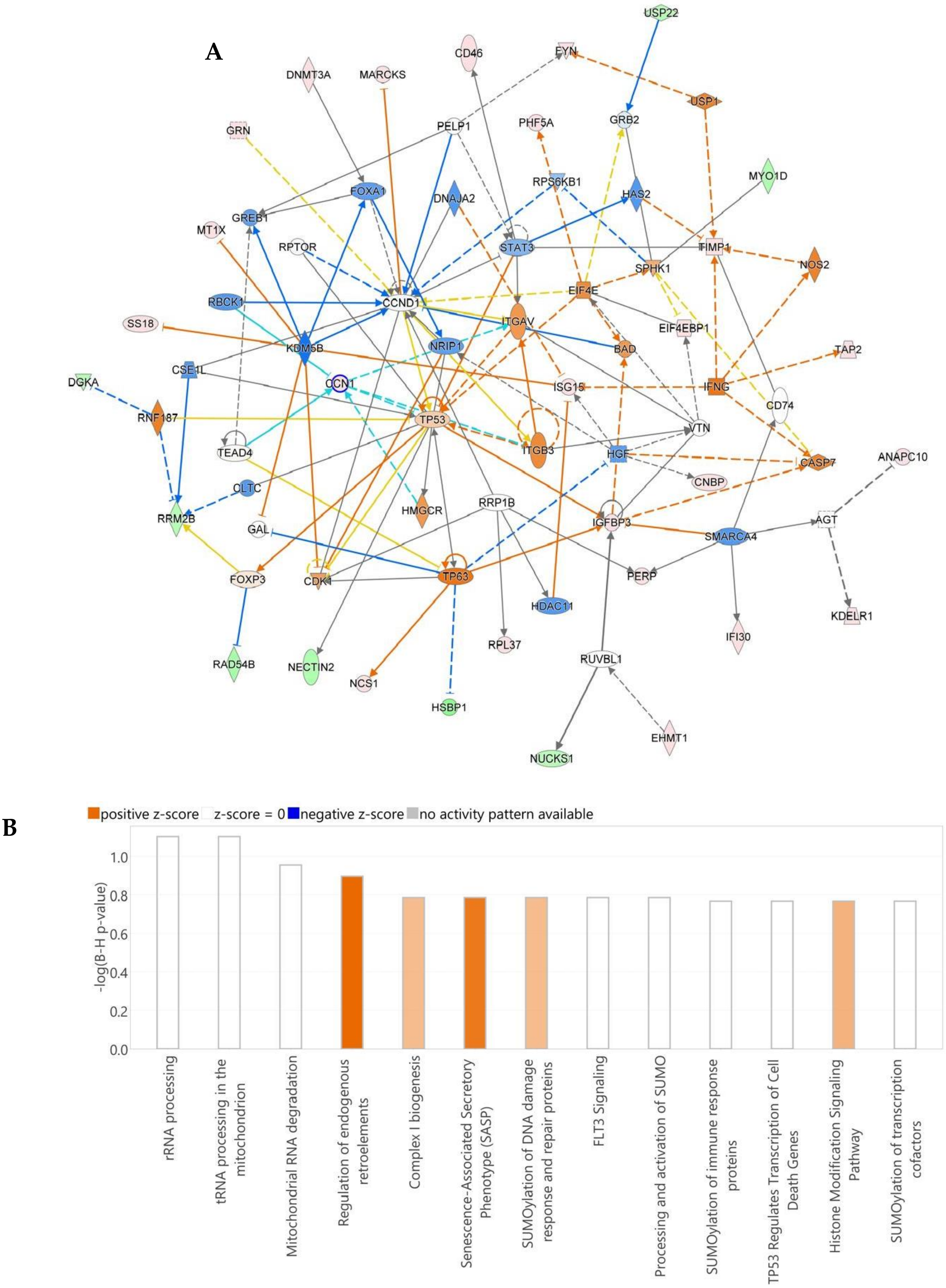

Supplementary Figure S4. (**A**) Network pathways derived from differentially regulated proteins in 400 μg/mL versus 200 μg/mL nanoceria-treated samples. Green nodes indicate downregulated proteins, and red nodes indicate upregulated proteins. (**B**) The most significant canonical pathways ranked by p-value, which were used to generate interaction networks.

Supplementary Table S1. Primer sequence.

Gene	**Forward Primer**	**Reverse Primer**	**Accession umber**
MnSOD	ACCACAGGCCTTATTCCACT	TACAACAGCTCAGCCACAGT	Y00497
CAT	TCCCAGAAGCCTAAGAATGCA	GCGATGATTACTGGTGAGGCT	NM_012520
GPx	CAGTCCACCGTGTATGCCTT	TGCCATTCTCCTGATGTCCG	NM_030826
SOD-2	ACCACAGGCCTTATTCCACT	TACAACAGCTCAGCCACAGT	XM_032894729.1
NF-kB	TGAGTCCCGCCCCTTCTAA	TGATGGTCCCCCCAGAGA	NM_00127671
BAX	GCACTAAAGTGCCCGAGCTG	GGGGGTCCCGAAGTAGGAAAG	NM_017059.2
BCL-2	CTGGCATCTTCTCCTTCCAG	CGGTAGCGACGAGAGAAGTC	NM_016993.2
BNIP3	CTCCTTTGCGGAGCCACCAT	GAGACGGAAGCTGGAACGCT	NM_053420.3
TFAM	TGTCATTGGGATTGGGCACA	AGATGCACGCACAGTCTTGA	XM_032888687
RF1	CATGGCCCTTAACAGTGAAGC	TGGTCCATGCATGAACTCCA	NM_001100708
PGC-1α	ACTCAGCAAGTCCTCAGTGC	TTCTGGTGCTGCAAGGAGAG	NM_031347
CDK4	TGGCACTTACACCCGTGGTT	TCTGGAGGCAGCCCAATCAG	NM_000075.4
P21	ATGTCAGAACCGGCTGGG	TCCCCAGCCTCTGACAT	L47233.1
P53	GCAGTCACAGCACATGACGG	CCAACCTCAGGCGGCTCATA	KY907141.1
Cyclin D1	TCTGTCGTGATGGGGCAAGG	GAAATGCACAGACCCAGCCG	BC023620.2
β-actin	CAACGTCACACTTCATGATGGA	ATGCCCCGAGGCTCTCTT	XM_032887061
